# TermGenie – a web-application for pattern-based ontology class generation

**DOI:** 10.1186/2041-1480-5-48

**Published:** 2014-12-11

**Authors:** Heiko Dietze, Tanya Z Berardini, Rebecca E Foulger, David P Hill, Jane Lomax, David Osumi-Sutherland, Paola Roncaglia, Christopher J Mungall

**Affiliations:** Genomics Division, Lawrence Berkeley National Laboratory, 1 Cyclotron Road, Berkeley, CA 94720 USA; The Arabidopsis Information Resource, Phoenix Bioinformatics, Redwood City, CA 94063 USA; European Molecular Biology Laboratory, European Bioinformatics Institute (EMBL-EBI), Hinxton, Cambridge CB10 1SD UK; Mouse Genome Informatics, The Jackson Laboratory, Bar Harbor, ME 04609 USA

**Keywords:** Ontology, Class generation

## Abstract

**Background:**

Biological ontologies are continually growing and improving from requests for new classes (terms) by biocurators. These ontology requests can frequently create bottlenecks in the biocuration process, as ontology developers struggle to keep up, while manually processing these requests and create classes.

**Results:**

TermGenie allows biocurators to generate new classes based on formally specified design patterns or templates. The system is web-based and can be accessed by any authorized curator through a web browser. Automated rules and reasoning engines are used to ensure validity, uniqueness and relationship to pre-existing classes. In the last 4 years the Gene Ontology TermGenie generated 4715 new classes, about 51.4% of all new classes created. The immediate generation of permanent identifiers proved not to be an issue with only 70 (1.4%) obsoleted classes.

**Conclusion:**

TermGenie is a web-based class-generation system that complements traditional ontology development tools. All classes added through pre-defined templates are guaranteed to have OWL equivalence axioms that are used for automatic classification and in some cases inter-ontology linkage. At the same time, the system is simple and intuitive and can be used by most biocurators without extensive training.

## Background

Biological ontologies such as the Gene Ontology (GO) and the Human Phenotype Ontology (HP) provide a rich set of constructs for describing biological entities such as genes, alleles and diseases. Like most data resources, ontologies are rarely complete, and healthy ontologies are continually growing and improving, as the state of knowledge progresses. One process by which ontologies grow is from requests for new classes (terms) by biocurators. These ontology requests can frequently create bottlenecks in the biocuration process, as ontology developers struggle to keep up with a deluge of requests.

**Figure 1:**
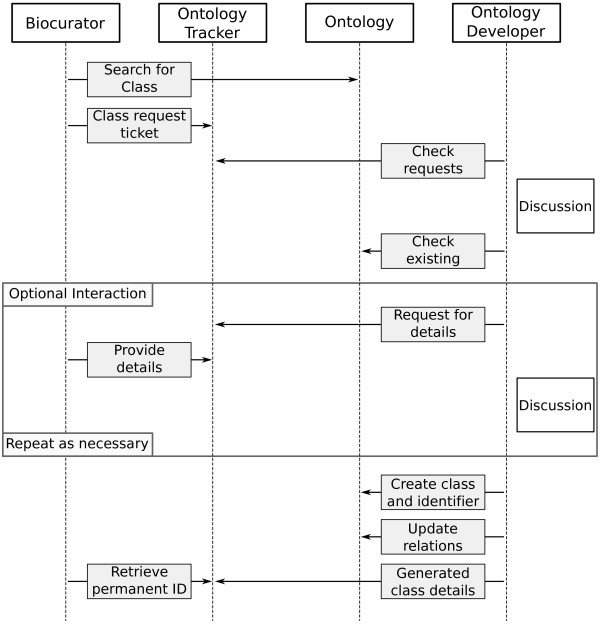
**Conventional ontology class request workflow.** General workflow for ontology class requests using a traditional issue tracker. A simple class request may take several days, for complex cases even longer.

Historically the process used in projects such as the GO Consortium would be for ontology developers to work through a set of requests collected in an issue tracking system, and to manually add them to the ontology, using a specialized Ontology Development Tool (ODT) such as OBO-Edit [1] – see Figure 1. Sometimes the ontology developers apply documented design patterns to guide this process, particularly where collections of classes follow a common structure. For example, most classes in the developmental process portion of the GO follow a consistent lexical form and relational structure as dictated in the GO developers documentation [2]. However, even with this documentation in place, this has still largely been a time-consuming and error-prone manual process, especially where ontology developers need to rearrange to the ontology structure.

Use of the Web Ontology Language (OWL), and in particular providing computable definitions in the form of equivalence axioms can greatly assist in ontology development and maintenance through the use of OWL reasoners. However, reasoners do not in themselves help with the task of class generation. Furthermore, for many biological ontologies, the axioms necessary for reasoning have been added post-hoc [3, 4] rather than prospectively at the time of class creation. This kind of retrospective axiomatization is inefficient but has in part been dictated by limitations of OBO-Edit. This can be partly circumvented by using an ODT that fully supports OWL such as Protégé, but this tool can be difficult for biocurators to use, and even in the hands of experts it can be time consuming to generate new classes complete with axioms referencing external ontologies.

Here we describe an application called TermGenie that allows biocurators to generate new classes based on formally specified design patterns or templates. The system is web-based and can be accessed by any authorized curator through a web browser. Automated rules and reasoning engines are used to ensure validity, uniqueness and relationship to pre-existing classes. The system makes extensive use of OWL axioms, but can be easily used without understanding these axioms. TermGenie is used extensively in the GO and is currently also in use for the Cell Type Ontology and for phenotype ontologies.

## Implementation

To minimize the entrance barrier for biocurators and non-experts, we provide TermGenie as a web application. The only requirement is a JavaScript enabled web browser. There are separate interfaces for separate tasks in TermGenie, one for class requests, and another for request review by ontology developers.

### Architectural components

**Figure 2:**
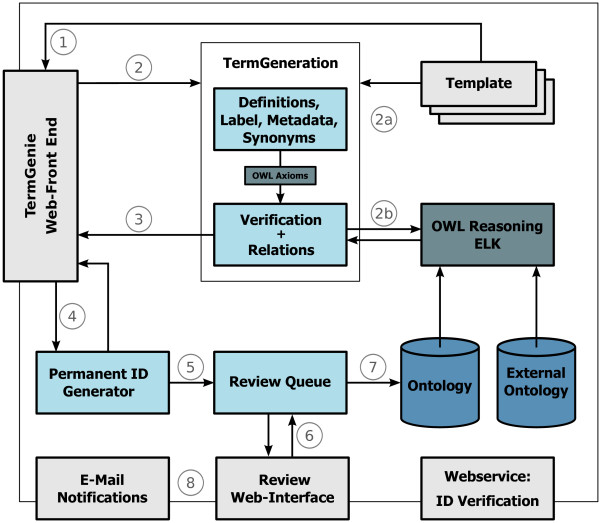
**Overview of TermGenie Components and Workflow.** (1) Retrieve existing templates for user selection; (2) Term generation processing and validation; (2a) Generate textual data and OWL axioms; (2b) Use reasoning to check for existing classes and new or changed relations; (3) Review of generated classes by the user in the web interface; (4) After review, assign permanent identifiers to the new classes; (5) Add the new classes into the queue for review; (6) Senior ontology developers review the classes: accept, modify, obsolete; (7) Commit the changes to the ontology; (8) Send confirmation e-mail to the user.

The TermGenie application is based on client-server architecture. The web client uses two JavaScript libraries (jQuery [5] and jQuery UI [6]) to implement the user interface in the web browser. The server is written in Java and accepts JSON messages in AJAX RPC calls from the client via a Java servlet listener. Figure 2 illustrates the required TermGenie components and the general workflow for ontology class generation.

The TermGenie server uses a set of modules and components to provide the required services for TermGenie. This set includes modules for basic functionalities such as loading ontologies, a persistence layer, reasoning, identifier generation, access to version control systems, and sending e-mails. Some modules are used for more complex components, such as the term generation, request submission, and review interface.

#### Ontology loading and synchronization

For ontology loading and the in-memory model, TermGenie relies on the OWL-API [7]. This Java library provides an axiom-based ontology model with parsers and writers for many OWL serialization formats. In addition, we use a Java implementation for handling OBO format [8], which also executes the conversion to the OWL in-memory model from OBO format. Future versions of the OWL-API will integrate the OBO format library, removing the need for this extra step. An important TermGenie feature is the support of ontology file handling in a version control systems (VCS). Currently TermGenie supports Subversion [9] and future support for Git [10] is planned. In addition, for a more efficient load of imports and files not in a VCS, TermGenie uses a local file cache for ontology files. The caching duration is a configuration parameter of each TermGenie installation. TermGenie loads the required ontologies during the server start. To keep the ontologies up-to-date and in sync with the source file, TermGenie periodically updates the VCS files and reloads the ontologies.

#### Request queue and services

As shown in the workflow, TermGenie saves the requested classes for review in a queue. This request queue is separate from the ontology file and requires a separate persistent storage module. The persistence module is implemented via the Java Persistence API using OpenJPA [11] as object-relational mapper and HSQLDB [12] as a simple embedded database for storage on disk. This lightweight default implementation makes TermGenie independent from more complex database setups and configuration issues. Because TermGenie does not push the requests to the ontology until they are reviewed, TermGenie provides additional services to access information about the pending request. Option one, there is a separate TermGenie page, which list the currently pending and recently approved requests. This table is intended for users to quickly check their recent requests. Option two, there is a web service to query the status of requested class and whether it is an approved, pending, or unknown class identifier. This service is intended for the integration of TermGenie in curation tools. Currently Protein2GO [13] uses the service to verify the class identifiers and prevent curators from entering invalid identifiers, while still allowing the immediate usage of newly generated classes.

#### Sessions and user authentication

TermGenie uses Java servlets mainly as abstraction layer, but we make use of the built-in session handling mechanism. The session is used to store the relevant tokens for the authentication of users. For the authentication, TermGenie currently relies on Persona [14] as a lightweight service. Persona is a 3rd-party (non-profit and open source) protocol and service, which uses an e-mail address as primary identifier. It provides a convenient JavaScript client library and easy server-side calls for token verification. Once a TermGenie session has been authenticated, the authorization module uses the e-mail address as primary identifier to check whether the user has the appropriate permissions for the requested operation. TermGenie has different sets of permissions depending on the tasks. For example, the submission of classes requires a different set of permissions than the TermGenie management console for administrators.

#### Logic-based autocompletion

An important convenience feature for TermGenie users is autocompletion. TermGenie uses a Lucene in-memory index to provide appropriate suggestions. To optimize the suggested classes and restrict the classes for a template, TermGenie can be configured to use only a subset of all available classes. For example, to create a subset for the molecular functions in GO, the configured set just contains the root class GO:0003674 (molecular_function). Using a reasoner, this set is then extended to include all direct and indirect subclasses. The same configuration mechanism can also be used to allow the input of classes from multiple ontologies in an input field. For example, it is possible to use cell-type classes form the Cell Type Ontology and plant cell classes from the Plant Ontology.

#### Configuration

All the different TermGenie components and modules are configured and combined via Google Guice [15], a lightweight dependency injection framework. TermGenie uses a combination of Java-based and compiler-checked defaults, configuration property files, and optional command-line overrides to configure a specific TermGenie installation. For example, the Guice modules are part of the Java configuration, creating a generic web application. The machine-specific details and secrets (e.g. passwords and private keys) are provided as a property file to override the default parameters. The location of the property file is declared via a command-line parameter. This helps to avoid the problem of accidental release of sensitive information into a public version control system.

#### Templating system

The core of TermGenie is template-based class generation. The template-based approach allows the separation of ontology design tasks, a fairly involved process, and standard class generation, a relatively straightforward task. For the former, the ontology developers extract or create, and test appropriate patterns for the generation of new classes based on the design principles of the ontology. A template consists of the OWL equivalent class axiom for the formal definition and reasoning, label and textual definition building blocks and, if applicable, details for synonym generation. These templates can then be used by biocurators to generate desired standard classes without need for knowledge of the internal workings of the ontology.

In TermGenie each template is specified as a separate JavaScript function and file. During the generation the JavaScript code is executed by a Java-embedded JavaScript engine. The embedded approach allows the use of native Java objects and functions, such as the ontology model and reasoner checks, in Javascript calls without the need for conversions. The Java layer also provides a set of functions intended to be used in the JavaScript code. These are shortcut functions for common tasks, such as the retrieval of a label for a given class. With these helpers it is possible to create fairly compact JavaScript code for a template. This approach does not preclude the application to more complex operations and checks. Most of the validation, such as the search for existing classes and the inference of relations, is done in Java using standard OWL-API reasoners.

**Figure 3:**
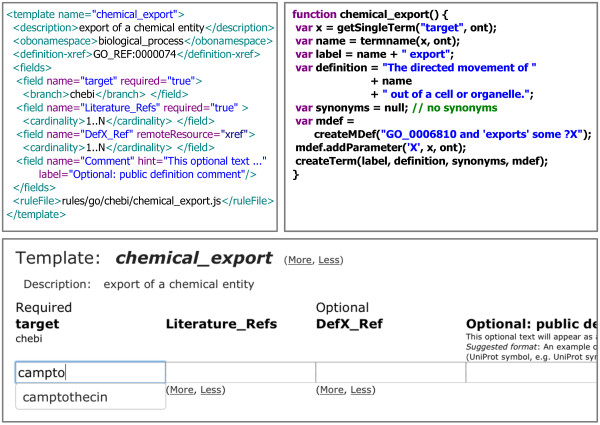
**Example template and configuration for TermGenie.** (top-left) XML-based example template configuration for the Gene Ontology template chemical_export. Includes declarations for required and optional input fields and corresponding JavaScript file; (top-right) Javascript snippet from the JavaScript file. for generating a class and OWL axioms; (bottom) Screenshot of the generated TermGenie input fields. Also shows autocompletion on ChEBI classes.

Every template has an associated XML-based configuration file. Amongst others, this configuration specifies the required and optional input fields, including details on the relevant ontology subsets for the appropriate auto-complete suggestions. For an example of a template with its XML configuration, JavaScript code and resulting input fields in TermGenie, see Figure 3. It should also be noted that this particular example template is configured to require exactly one ontology class as input. Other templates can use up to three different input classes in the equivalent class axioms for a generated class.

#### Reasoning

**Figure 4:**
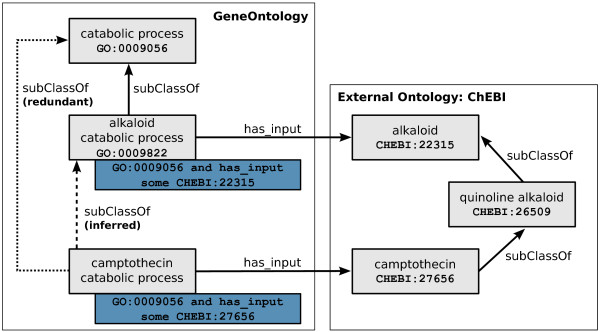
**Inferences for a class using a standard OWL reasoner.** Reasoning example for a *genus* + *differentia* pattern for camptothecin catabolism in the GeneOntology. The class is defined by its *genus* ‘catabolic process’ (GO:0009045) and *differentia* ‘has_input camptothecin’ (CHEBI:27656). Following that definition, the class is a subclass of catabolic process. Using the additional axioms from ChEBI and the GeneOntology, a standard OWL reasoner can infer the more specific superclass ‘alkaloid catabolic process’ (GO:0009822).

TermGenie uses reasoning for two tasks: validation and relation inference. Both tasks rely on the equivalent class axioms specified in the templates. For the validation, TermGenie asks the reasoner for equivalent named classes for the given hypothetical new class. Similarly, for the inference and update of relations, we query the reasoner for the direct super- and subclasses of the hypothetical class. This is done by declaring a new class using a new temporary identifier and adding the corresponding equivalent class axioms. Next, TermGenie creates an up-to-date reasoner instance for the changed ontology. To prevent unpredictable inferences, the ontology is checked for inconsistency and unsatisfiable classes. Once these checks are completed, the actual new-class-related queries are done. After querying, the axiom changes are reverted and the reasoner is discarded. The inferred direct subclasses are used to assert the most specific superclasses. In addition the direct subclasses of the hypothetical new class are checked and their relations updated. This strategy allows the creation of not only the new leaf classes in the ontology graph, but also new intermediate classes with an automatic update of relations for existing classes. An example of an inference using equivalent class axioms and a reference ontology is available in Figure 4.

Using this workflow the reasoner creation and querying are the most time-consuming steps of a TermGenie template request. In theory, TermGenie can use any OWL-API compliant reasoner, but the requirements for an interactive web-application introduce a processing time limit for the reasoner. We have experimented with multiple reasoners and chose ELK [16] as the most convenient compromise for TermGenie.

### User workflow

**Figure 5:**
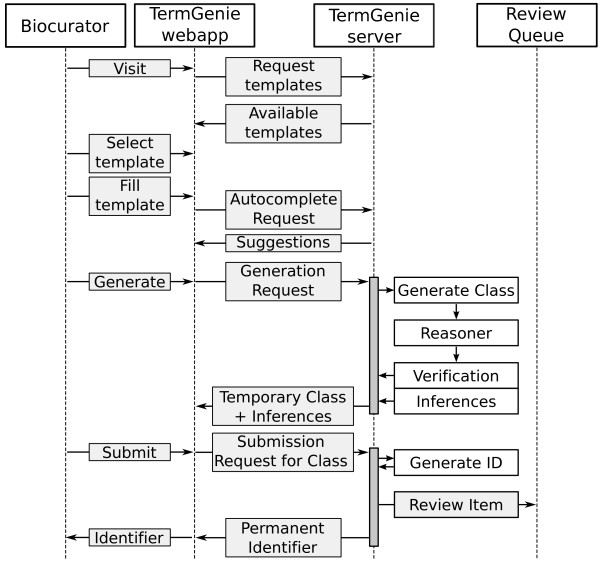
**TermGenie user workflow.** To create a class in TermGenie, Biocurators go to the TermGenie website and select the relevant template for their request. The template consists of a set of required and optional input fields. TermGenie provides autocompletion for appropriate input fields. After passing some quick checks, the request is sent to the server, where generation and reasoning are executed. The results are send back and the users have the chance to review the proposed classes. The next step is the submission of the generated classes for review. As part of this process, a new permanent identifier is generated using a customizable identifier pattern and range. Furthermore, the request is added to the review queue for final approval by the ontology developers.

In a typical workflow, the user begins by loading the relevant TermGenie web page, selecting and filling in the relevant template. After the class generation and validation, the class is submitted for user review and approval and assignment of a permanent identifier, see also Figure 5 for a workflow diagram.

On the user side, a TermGenie template consists of a set of required and optional input fields. TermGenie provides autocompletion for ontology classes for appropriate input fields. Before a user starts the new class generation and validation, a number of quick checks are executed. The checks include one for missing entries in required input fields, such as a missing literature reference. After passing these checks, the request is sent to the server. After the generation and reasoning step on the server, the users have the chance to review the proposed classes. They can also make modifications to textual parts if necessary (e.g. definition) or add additional synonyms. The next step is the submission of the generated classes for review. As part of this process, a new permanent identifier is generated using a customizable identifier pattern and range. To complete the submission step, the user must be logged in (authenticated), as the server will check for the appropriate permissions and will use the user metadata for provenance information of the generated classes and requested e-mail notifications.

Ideally, after generating the identifier, a biocurator can immediately use the generated identifiers for annotation. To facilitate this even while the identifier is not yet committed to the ontology, we provide a web service to check the validity of class identifiers.

### Review process

After a user has submitted their generated class requests and generated the permanent identifiers, the requests are put into a queue for review by an ontology developer. During the review the ontology developer has the following three choices: approve, modify, or obsolete. There is no reject or delete option at that stage because a permanent identifier has already been generated. In most cases the classes can be approved without (or with minimal) modifications since they rely on tested templates. Should a developer need more details, he/she may contact the original requester without making a decision, and keep the request pending. The ontology developers can use the e-mail information available from the provenance information of each request.

**Figure 6:**
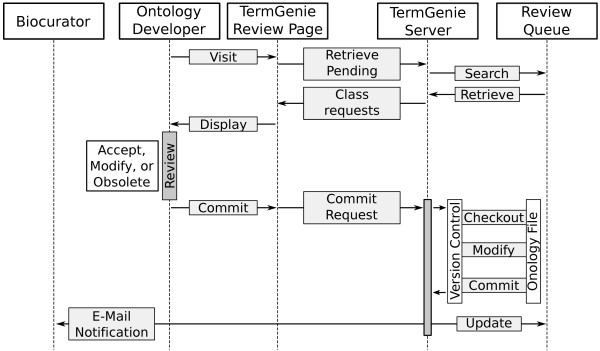
**TermGenie workflow during a submitted class review by an ontology developer.** After a user has submitted their generated class requests, the requests are put into a queue for review by an ontology developer. During the review the ontology developer has the following three choices: approve, modify, or obsolete. For the commit, the server uses the version control adapter to create a clean checkout. From there TermGenie loads the ontology as a separate instance and applies the relevant changes. After writing the changed ontology as a file, TermGenie tries to commit the updated file into the version control. After a successful commit the queue is updated and a confirmation e-mail is sent to the requester.

Once the ontology developer has determined which classes to commit to the ontology, he/she selects the corresponding checkboxes and initiates the commit. On the server, the classes from the requests are first quickly checked again. For the commit, the server uses the version control to create a clean checkout. From there TermGenie loads the ontology as a separate instance and applies the relevant changes. Depending on the original ontology file format this can either be OWL axioms or OBO term frames. After writing the changed ontology as a file, TermGenie tries to commit the updated file into the VCS. When there is more than one request selected for commit to the ontology, TermGenie processes each one separately with individual commits. This allows for a more fine-grained and aspect-oriented tracking of changes in the underlying VCS. See also Figure 6 for the workflow during the review process.

## Results and discussion

**Usage in the Gene Ontology** The pattern-based TermGenie approach has been used for the Gene Ontology since July 2010, with the current Java implementation in place since November 2012. During the period from July 2010 until the end of June 2014, the Gene Ontology instance of TermGenie has been used to generate 4715 classes, which represents 51.4% of all new classes created in GO during that time. For a quarterly report of new classes in GO see Table 1. The number of available templates has been growing over time and currently stands at 38 templates, see also Table 2 for a list of the templates. Many of these templates utilize an external ontology.

**Table 1:** **TermGenie generated class counts in GO over time**

Quarter	2010-III	2010-IV	2011-I	2011-II	2011-III	2011-IV	2012-I	2012-II	
TermGenie	139	154	236	254	307	175	255	806	
Manual	575	413	332	295	313	364	462	324	
Fraction	19.47%	27.16%	41.55%	46.27%	49.52%	32.47%	35.56%	71.33%	
**Quarter**	**2012-III**	**2012-IV**	**2013-I**	**2013-II**	**2013-III**	**2013-IV**	**2014-I**	**2014-II**	**Total**
TermGenie	303	352	357	285	218	231	301	342	4715
Manual	371	283	62	92	170	164	109	110	4439
Fraction	44.96%	55.43%	85.20%	75.60%	56.19%	58.48%	73.41%	75.66%	51.51%

**Table 2:** **Available templates for the geneontology termgenie instance**

Template	Input fields	Equivalent class statement
**regulation: biological process**		
regulation	X:BP	GO:0065007 and ‘regulates’ some ?X
negative_regulation	X:BP	GO:0065007 and ‘negatively regulates’ some ?X
positive_regulation	X:BP	GO:0065007 and ‘positively regulates’ some ?X
**regulation: molecular function**		
regulation	X:MF	GO:0065007 and ‘regulates’ some ?X
negative_regulation	X:MF	GO:0065007 and ‘negatively regulates’ some ?X
positive_regulation	X:MF	GO:0065007 and ‘positively regulates’ some ?X
involved_in	P:BP, W:BP	?P and ‘part_of’ some ?W
involved_in_mf_bp	P:MF, W:BP	?P and ‘part_of’ some ?W
occurs_in	P:BP, C:CC	?P and ‘occurs in’ some ?C
regulation_by	R:GO:0050789, P:BP	?R and ‘results_in’ some ?P
part_of_cell_component	P:CC, W: CC	?P and ‘part_of’ some ?W
chemical_transport	X:chebi	GO:0006810 and ‘transports or maintains localization of’ some ?X
chemical_transporter_activity	X:chebi	GO:0005215 and ‘transports or maintains localization of’ some ?X
chemical_binding	X:chebi	GO:0005488 and ‘has input’ some ?X
**metabolism_catabolism_biosynthesis**		
metabolism	X:chebi	GO:0008152 and ‘has participant’ some ?X
catabolism	X:chebi	GO:0009056 and ‘has input’ some ?X
biosynthesis	X:chebi	GO:0009058 and ‘has output’ some ?X
chemical_transmembrane_transport	X:chebi	GO:0055085 and ‘transports or maintains localization of’ some ?X
**chemical_transmembrane_transporter_activity**		
transmembrane transporter activity	X:chebi	GO:0022857 and ‘transports or maintains localization of’ some ?X
secondary active transmembrane transporter activity	X:chebi	GO:0015291 and ‘transports or maintains localization of’ some ?X
uptake transmembrane transporter activity	X:chebi	GO:0015563 and ‘transports or maintains localization of’ some ?X
transmembrane-transporting ATPase activity	X:chebi	GO:0042626 and ‘transports or maintains localization of’ some ?X
**chemical_response_to**		
response to	X:chebi	GO:0050896 and ‘has input’ some ?X
cellular response to	X:chebi	GO:0070887 and ‘has input’ some ?X
**chemical_homeostasis**		
chemical homeostasis	X:chebi	GO:0048878 and ‘regulates level of’ some ?X
cellular chemical homeostasis	X:chebi	GO:0055082 and ‘regulates level of’ some ?X
chemical_import	X:chebi	GO:0006810 and ‘imports’ some ?X
chemical_export	X:chebi	GO:0006810 and ‘exports’ some ?X
chemical_import_into	S:chebi, T:CC	GO:0006810 and ‘has target end location’ some ?T and ‘imports’ some ?S
**cc_transport_from_to**		
transport	F:CC, T:CC	GO:0006810 and ‘has target start location’ some ?F and ‘has target end location’ some ?T
vesicle-mediated transport	F:CC, T:CC	GO:0016192 and ‘has target start location’ some ?F and ‘has target end location’ some ?T
**cc_transport**		
transport	C:CC	GO:0006810 and ‘transports or maintains localization of’ some ?C
vesicle-mediated transport	C:CC	GO:0016192 and ‘transports or maintains localization of’ some ?C
**chemical_transport_from_to**		
transport	X:chebi, [F:CC], [T:CC]	GO:0006810 and ‘transports or maintains localization of’ some ?X [and ‘has target start location’ some ?F] [and ‘has target end location’ some ?T]
vesicle-mediated transport	X:chebi, [F:CC], [T:CC]	GO:0016192 and ‘transports or maintains localization of’ some ?X [and ‘has target start location’ some ?F] [and ‘has target end location’ some ?T]
**cc_assembly_disassembly**		
assembly	C:CC	GO:0022607 and ‘results_in_assembly_of’ some ?C
disassembly	C:CC	GO:0022411 and ‘results_in_disassembly_of’ some ?C
plant_development	P:plant	anatomical structure development’ and ‘results in development of’ some ?P
plant_formation	X:plant	anatomical structure formation involved in morphogenesis’ and ‘results in formation of’ some ?X
plant_maturation	X:plant	developmental maturation’ and ‘results in developmental progression of’ some ?X
plant_morphogenesis	X:plant	anatomical structure morphogenesis’ and ‘results in morphogenesis of’ some ?X
plant_structural_organization	X:plant	anatomical structure arrangement’ and ‘results in structural organization of’ some ?X
cell_apoptotic_process	C:cell	cell-type specific apoptotic process’ and ‘occurs in’ some ?C
cell_differentiation	C:cell	GO:0030154 and ‘results in acquisition of features of’ some ?C
cell_migration	C:cell	cell migration’ and ‘alters location of’ some ?C
**protein_localization_to**		
protein localization	C:CC	GO:0008104 and ‘has target end location’ some ?C
establishment of protein localization	C:CC	GO:0045184 and ‘has target end location’ some ?C
protein_complex_by_activity	A:MF	GO:0043234 and ‘capable_of’ some ?A
**single_multi_organism_process**		
single-organism	P:BP	?P and ‘bearer of’ some PATO:0002487
multi-organism	P:BP	?P and ‘bearer of’ some PATO:0002486
biosynthesis_from	T:chebi, F:chebi	GO:0009058 and ‘has output’ some ?T and ‘has input’ some ?F
biosynthesis_via	T:chebi, V:chebi	GO:0009058 and ‘has output’ some ?T and ‘has intermediate’ some ?V
catabolism_to	S:chebi, R:chebi	GO:0009056 and ‘has input’ some ?S and ‘has output’ some ?T
catabolism_via	X:chebi, V:chebi	GO:0009056 and ‘has input’ some ?X and ‘has intermediate’ some ?V
metazoan_development	X:Uberon	anatomical structure development’ and ‘results in development of’ some ?X

The first column contains the template names and available templates variations. The second column lists the expected ontology inputs for the equivalent class statement in the third column, with BP = GO:biological_process, MF = GO:molecular_function, CC = GO:cellular_component, chebi = ‘chemical entity’ (CHEBI:24431), plant = ‘plant anatomical entity’ (PO:0025131), cell = ‘native cell’ (CL:0000003), Uberon = ‘anatomical entity’ (UBERON:0001062).

As described before, TermGenie relies heavily on reasoning for automatic classification and validation. This requires that the ontology underlying a TermGenie instance be sufficiently axiomatized with equivalent class axioms. In the case of the Gene Ontology, with its considerable size and development history, a significant amount of time and effort was needed to introduce equivalent class axioms into the ontology. The formalization of GO started in the early 2000s [17] and is still an ongoing task. It not only includes intra-ontology definitions [18], but also makes use of existing other domain-specific ontologies, such as the Chemical Entities of Biological Interest (ChEBI) ontology [19]. The most frequently referenced external ontology is ChEBI, but we also use the Plant Ontology (PO), Cell Type Ontology, Phenotypic Quality Ontology (PATO), and Uberon [4] to define class patterns in GO. One could argue that ontology formalization is critical in creating a scalable and affordable long-term maintenance strategy because it supports automatic inferences and reasoning. The template-based formalization process helps to make implicit design patterns and assumptions explicit.

**Streamlining ontology development** The template-based approach allows the separation of concerns and roles between ontology engineering and everyday ontology class requests. Most of the ontology work for creating a template can be done by the ontology developers and OWL experts during the design and test phase for each of the templates. Once a pattern has been created and is available in a TermGenie web application, adding a new class in that same pattern is vastly streamlined. The biocurators can quickly and safely create classes and permanent identifiers on the website within minutes and return to their annotation task. The effort for the final review by an ontology developer for each class in TermGenie is minimal as it relies on a pre-existing and tested solution. TermGenie also provides the convenient feature of e-mail notifications.

**Bounds on complexity of composed classes** Even though templates are usually tested and approved by the ontology developers, one interesting issue for Gene Ontology requests has come up. Some templates generate classes of the same category as the input class (e.g. process involved_in process, or regulation of processes). This means that it is possible to recursively compose classes with definitions that unfold to a deeply nested hierarchy, with complex textual definitions and labels that impose a cognitive burden on users. Most of these classes are requested for the annotation of complex biological processes and functions with a pre-composition strategy or legacy systems with a simplistic annotation model (e.g. single unrelated annotations). From the formal point of view these classes have a clear axiomatized definition and can be unfolded into simpler annotations [20]. This kind of class request, although not very common in TermGenie, take longer to review as they often require further discussion and modifications by ontology developers. One proposal has been to design a strategy to prevent the creation of these multiply compounded classes and to instead redirect users to the issue tracker instead of proceeding with the request. The detection and redirection feature has not yet been implemented.

The most time-saving feature for biocurators is the immediate creation of permanent identifiers. Therefore, during the review by an ontology developer, this leaves only obsoletion as a way to reject a request. In theory this could lead to higher number of unnecessary obsoleted classes. However, this proved not to be an issue for the Gene Ontology TermGenie instance. Only 70 requested classes have been obsoleted since inception, about 1.6% of the TermGenie requests.

**Non-templated class generation** Biology and other complex subjects cannot always be axiomatized in a templatable way. Therefore, not all class requests can or should be done using a template. To address this issue and at the request of the ontology developers, we added a free-form option to TermGenie. This allows very experienced users to quickly specify all the relevant details of a class, validate, and generate the new class using TermGenie. The free-from workflow extends to the existing validation procedures with additional checks. It searches for and warns about existing similar class names and synonyms for a given class request. For example, a request for ‘omegasome’ via free-form, produces a warning that a similar class ‘megasome’ already exists. In this case the warning could be dismissed as the two classes refer to completely different cell components. This additional check helps the ontology developers to avoid the creation of redundant classes.

Due to the different use-case, this free-form template is implemented as a separate tool in the TermGenie webapp, but shares many services (e.g., autocomplete, e-mail notifications) and adds requests to the common review queue. Furthermore, we use a different set of permissions to restrict the access of users to this template. Due to the more experimental nature of the requests via the free-form template, the obsoletion rate is slightly higher, with 16 of 387 (4.1%) obsoleted requests.

**Evaluation of OWL reasoners for use in TermGenie** Because reasoning is a core task in TermGenie, we experimented with multiple OWL-API compliant reasoners. Currently, we haven chosen ELK [16] as the best compromise for TermGenie. ELK is an OWL 2 EL profile [21] reasoner and provides a good trade off between response time and supported inference. Other tested reasoners include HermiT [22], JFact [23], Pellet [24], MORe [25] as full OWL compliant reasoners and jcel [26] as another OWL 2 EL compliant reasoner. In general all full OWL2 reasoners proved to be too slow for usage in TermGenie. The other EL reasoner, jcel, is a viable alternative, but ELK using multithreading out-performed jcel in the initial classification step. A typical reasoning task for the Gene Ontology and the required external ontologies includes about 415,000 logical axioms. Using ELK, we can respond to a single request within a few seconds.

**TermGenie for other ontologies** The TermGenie system was designed from the outset to be ontology-neutral. In addition to the Gene Ontology instance [27], we have worked with the developers of other OBO Library ontologies to create custom TermGenie instances.

The OBO Cell Type Ontology (CL) [28] represents cell types found in animals. One of the main uses of the CL is to rigorously describe samples collected as part of large next-generation sequencing projects such as Functional Annotation of Mammalian Genomes 5 (FANTOM5) and the Encyclopaedia of DNA Elements (ENCODE), allowing analyses that yield insight into properties of different cell types [29]. The ENCODE curators have found the CL instance of TermGenie useful as it provides a simple web-based way to generate new classes used to describe samples.

The Ontology of Biological Attributes (OBA) [30] was created as a unified representation of traits (for example ‘eye color’) encompassing ontologies for describing animals, plants and single-celled organisms. Many traits follow a trivial compositional pattern, encompassing a simple entity-attribute pattern, with the attribute being taken from the ‘attribute’ subset of PATO, and the entity taken from ontologies such as Uberon or PO. This ontology was originally created to be able to structure the ‘regulation of biological quality’ branch of the GO, but it has found uses in other areas. Curators in the Monarch Initiative project have used it to describe mouse strain phenotypes, and most recently it has incorporated into the Encyclopaedia of Life (EOL) TraitBank [31] project.

Ontologies of abnormal or variant phenotypes also benefit from a templated approach, as their classes can often be described using an Entity-Quality combinatorial approach, akin to that used in OBA. So far we have created instances for the Mammalian Phenotype Ontology [32] and the Human Phenotype Ontology (HP) [33], with plans to create instances for other species-specific phenotype ontologies. The HP instance was created in part to serve the needs of the NIH Undiagnosed Diseases Program (UDP), which is systematically describing the phenotypes of patients with undiagnosed diseases, so that phenotype comparison algorithms can be used to assist the hunt for the genomic underpinnings of these diseases. In this case, it is important that a diversity of ontology contributors can efficiently and effectively contribute to the HP. We believe that TermGenie will greatly facilitate contributions from the rare disease community.

Note that in order for these instances to work effectively, it was first necessary for the respective developers to make their ontologies ‘reasoner-ready’ by providing OWL equivalent class axioms, a process that has been underway for several years [3, 34]. In contrast, once the necessary OWL refactoring is complete, the configuration of the ontology-specific TermGenie instances takes about a week, with most of the time spent on testing the templates.

### Comparison with other approaches

Creating new classes in ontologies is a common task, one that is typically done by a developer using an Ontology Development Tool (ODT) such as OBO-Edit [1] or Protégé [35]. These are both comprehensive, general purpose environments, and are not intended for use by annotators and biocurators without requisite training. In addition, both are desktop applications, requiring an installation on the user’s machine. The limitations of desktop ontology development software, especially for collaborative work, led to the creation of WebProtégé, a web-based ontology development tool [36]. All three applications are powerful tools with steep learning curves and are usually intended for knowledge/ontology engineers and ontology developers. They do not offer the separation of design and quick everyday use for non-experts. TermGenie is not intended to replace comprehensive ODTs; the pattern-based approach and ODTs complement each other in the ontology development workflow. In fact the comprehensive ODTs are required during the template design and testing.

Other related work exists in the form of the Term Generation plugin DOG4DAG [37]. It is available as an OBO-Edit and Protégé plugin. The tool allows proposal of new classes based on phrases extracted from a given text corpus. The most common use case is to create or add domain-specific vocabulary to an ontology. The best use is in early stages of ontology projects as it generates mostly list of candidate classes. For a more mature and formalized ontology, a more axiomatized result is required.

The Network-Extracted Ontology (NeXO) [38] is an example of an orthogonal, data-driven approach to ontology generation. This approach takes as input a sufficiently large and dense network (i.e. gene and protein interactions), and applies a clustering algorithm to generate classes and relationships between these classes. So far, NeXO has been used to generate a yeast cellular component ontology. It remains to be seen how well the approach works for other portions of ontologies such as the GO.

Another template-driven class generation approach is Quick Term Templates [39]. There are multiple implementations available for this approach: a MappingMaster plugin for Protégé, the OntoRat web application [40], or custom Perl code combined with spreadsheets. Each implementation still requires quite a bit of detailed knowledge of the ontology. One huge issue is the information flow back to the ontology developers, which also includes the assignment of valid/permanent identifier and access control. In the TermGenie application these details are controlled by the server and the built-in review mechanism.

The Cellular Phenotype Ontology (CPO) is an ontology that was entirely generated programmatically [41]. A custom program was written using the java OWL-API to generate a class from the cross-product of the ‘cellular process’ branch of GO and a subset of PATO. This kind of en-mass class generation is in contrast to the TermGenie approach, in which biocurators flesh out a subset of the space of all possible classes on an as-needed basis. The resulting ontology is more compact and is arguably more usable than one in which the entire space of terms is fleshed out in advance.

Most recently, the Tawny-OWL framework provides an elegant and powerful way to generate an entire ontology programmatically using a high-level declarative domain-specific language [42]. At this time, Tawny is difficult to integrate into a conventional ontology development workflow as it requires the source for the ontology to be stored as a Clojure program rather than in a non-programmatic format such as OBO or OWL. However, we are working with the Tawny developers to explore ways to integrate our approaches.

## Conclusion

TermGenie is a web-based class-generation system that complements traditional ontology development tools. All classes added through pre-defined templates are guaranteed to have OWL equivalence axioms that are used for automatic classification and in some cases inter-ontology linkage. At the same time, the system is simple and intuitive and can be used by most biocurators without extensive training. Its use in the Gene Ontology has removed a significant curation bottleneck, and has freed up ontology developers from performing time-consuming repetitive tasks allowing them to work on high-level design issues. In the last 4 years the Gene Ontology TermGenie instance was used to generated 4715 new classes, about 51.4% of all new classes created. The immediate generation of permanent identifiers proved not to be an issue with only 70 (1.4%) obsoleted classes. TermGenie is now in use in other projects as well, including the Mammalian Phenotype Ontology, the Human Phenotype Ontology, the Cell Type Ontology and the Ontology of Biological Attributes.

## Availability and requirements

- ● **Project name:** TermGenie
- ● **Project home page:**http://termgenie.org
- ● **Operating system(s):** Platform independent
- ● **Programming language:** Java, JavaScript
- ● **Other requirements:** Java 6 or higher, Jetty 6 or higher, Maven 3.0.x
- ● **License:** New BSD (BSD 3 Clause)
- ● **Any restrictions to use by non-academics:** none

## Abbreviations

GO: GeneOntology
HP: Human Phenotype
ODT: Ontology Development Tool
OWL: Web Ontology Language
VCS: Version Control System
ChEBI: Chemical Entities of Biological Interest
PO: Plant Ontology
CL: Cell Type Ontology
PATO: Phenotypic Quality Ontology
OBA: Ontology of Biological Attributes.
